# Assessing the feasibility of quantitative SPECT imaging for low ^212^Pb activity concentrations using anthropomorphic phantoms

**DOI:** 10.1186/s40658-025-00829-1

**Published:** 2026-01-05

**Authors:** Johan Høiness, Caroline Stokke, Eivor Hernes, Lars Tore Gyland Mikalsen, Monika Kvassheim

**Affiliations:** 1 Department of Physics, University of Oslo, Oslo, Norway; 2 Department of Physics and Computational Radiology, Division of Radiology and Nuclear Medicine, Oslo University Hospital, Oslo, Norway; 3 Department of Nuclear Medicine, Division of Radiology and Nuclear Medicine, Oslo University Hospital, Oslo, Norway; 4 Faculty of Medicine, University of Oslo, Oslo, Norway

**Keywords:** SPECT, ^212^Pb, Phantom, Anthropomorphic, Quantitative, 3D printing

## Abstract

**Background:**

Lead-212 (^212^Pb) is being investigated for alpha therapies, but it can be challenging to image. To investigate the quantitative accuracy of ^212^Pb SPECT images for patient geometries and low activity concentrations, we imaged an anthropomorphic phantom with ^212^Pb, and studied the deviations of SPECT derived activity concentrations.

**Methods:**

Fillable phantom compartment shells of the kidneys, liver and five vertebrae (T11-L3) were 3D-printed based on a patient’s CT-images. The same patient’s [^18^F]F-PSMA-1007 PET image was used as a basis for the relative distribution of ^212^Pb activity within the phantom compartments. The phantom was imaged with a Siemens Symbia Intevo Bold SPECT/CT, with a total of 3.4–3.8 MBq ^212^Pb for three acquisitions and 1.0–1.1 MBq ^212^Pb for three acquisitions, while recording energy windows centred at 79 keV and 239 keV. The SPECT images were reconstructed with Siemens’ Flash-3D with a variety of iterations, subsets, and matrix sizes. Activity concentrations for each phantom compartment were measured from the images using a calibration factor measured in a uniform cross calibration phantom and compared to the activity concentrations in 1 ml samples extracted from each compartment, which were analysed using a gamma counter.

**Results:**

Quantification was relatively stable across energy windows and matrix sizes, but best results were achieved using 30–120 reconstruction updates. Low activity concentration volumes representing background and vertebral bodies (0.03–0.05 kBq/ml) were not quantifiable with deviations over 400% for all investigated reconstructions. The activity concentrations in the liver and kidneys were underestimated by 10–50% compared to the gamma counter measurements. Precision between SPECT acquisitions was higher for the larger image matrix, with standard deviations of liver and kidney measurements less than 6% for the higher activity images, and less than 8% for the lower activity images.

**Conclusion:**

We found that larger volumes, such as liver and kidneys with at least 210 Bq/ml, may be quantifiable with an accuracy of approx. 30–40%. While very low activity concentrations below 54 Bq/ml were not quantifiable, this still indicate carefully used imaging results to be of value in dosimetric calculations, also when characterising latter parts of time activity curves.

**Supplementary Information:**

The online version contains supplementary material available at 10.1186/s40658-025-00829-1.

## Introduction

Targeted Alpha Therapy (TAT) is considered challenging to image, due in part to the small amounts of activity administered [1]. For several nuclides, including ^212^Pb, the complicated SPECT energy spectrum further adds to this challenge [2]. ^212^Pb is itself a β-emitter with a half-life of 10.6 h, but is regarded as a TAT radionuclide due to its first daughter bismuth-212 (^212^Bi), which has a half-life of 61 min and decays either with α-emission to thallium-208, or by β-emission to polonium-212 which in turn rapidly decays with α-emission to lead-208. The decay chain of ^212^Pb features emissions of 238.6 keV gamma-rays (intensity 43.3%), a set of x-rays centred around 79 keV (total intensity 36%), and high-energy gamma-rays of 2614 keV (intensity 36%) [3]. The 75–88 keV and 239 keV emissions are suitable for SPECT imaging [3, 4], but only 6% and 3% of the signal recorded within a 40% energy window around 79 keV and 20% around 239 keV, respectively, originates from these emissions [2]. Downscatter and characteristic x-rays from interactions of the 2614 keV gammas in the collimator massively contribute to the energy spectrum on the SPECT and obscures the signal from the direct emissions in the captured energy range [2].

This study was performed in relation to a phase 0 trial where patients were administered 10 MBq of [^212^Pb]Pb-PSMA (AB001) [5], and our study intended to determine whether the low activity concentration patient SPECT images could be quantified to support dosimetric evaluations. The feasibility of quantitative ^212^Pb imaging has been studied in simple phantoms with promising uncertainties [3]; in this study we apply these results to patient geometries to investigate quantitative accuracy.

The aim of our study was to investigate the feasibility of accurate quantification of low activity concentrations of ^212^Pb. To this end, an anthropomorphic phantom, with 3D-printed compartments containing a liver, a pair of kidneys, and T11–L3 vertebrae, was constructed and scanned at activity levels relevant to the phase 0 study [5].

## Methods

### Phantom design and construction

Based on patient CT images, anthropomorphic phantom compartments representing the liver, kidneys, and T11–L3 vertebrae were 3D-printed to replicate the patient-specific geometry. The organ shapes were segmented from CT images of the patient using 3D Slicer (v5.2.2–v5.6.1, https://www.slicer.org), partially assisted by TotalSegmentator [6]. The kidney and liver phantoms were modelled as 2 mm and 3 mm outer shells on their segmentations, respectively. The vertebrae segmentations were used to generate 2 mm inner shells, with additional 2 mm inner dividers to split the vertebral bodies and vertebral arches into their own compartments. These models were then exported to Autodesk Fusion 360 for further modifications, including the addition of filling ports and structural connector points.

The vertebrae were 3D-printed with higher-attenuation filament (DXTECH SimuBone™ Bone Simulation Modeling Filament) to mimic cortical bone, while the liver and kidneys were printed using polylactic acid (PLA) filament (Clas Ohlson By Flashforge). Fixation structures were printed in PLA to ensure accurate anatomical positioning of the phantom inserts. All components were printed whole using a modified Creality Ender-3 Pro 3D-printer, except for the liver which was printed in two parts using an UltiMaker S5. The liver was split along the axial plane, and 5 mm lips were added to join the halves using epoxy resin. The phantom compartments were sprayed with lacquer to increase water tightness. Three additional layers of epoxy resin were added on the liver compartment. The chambers in the vertebrae arches were filled with a solution of 70% water and 30% (by weight) dipotassium hydrogen phosphate (K_4_HPO_4_).

All phantom compartments were placed in a cylindrical container (inner diameter 380 mm, inner height 340), which was partially filled with approximately 28 L of water for imaging.

### Radionuclide distribution

The ^212^Pb used in this study was obtained from Institute of Cancer Research, Oslo University Hospital, which was produced from thorium-228 (Eckert & Ziegler, Braunschweig, Germany or Oak Ridge National Laboratory, Oak Ridge, TN, USA) as described by Li et al. [7]. The activity distribution in the phantom compartments was derived from a [^18^F]F-PSMA-1007 PET image of the patient. The phantom was filled twice. Initially, it was filled with 40.8 MBq of ^18^F as measured in the [^18^F]F-PSMA-1007 image and compared to the clinical image. The activity concentrations in left kidney, right kidney, liver, vertebral bodies, and background were 10.1 kBq/ml, 12.4 kBq/ml, 11.5 kBq/ml, 0.5 kBq/ml, and 0.4 kBq/ml, respectively, at the time of PET imaging. Subsequently, the phantom was filled with ^212^Pb, with the same relative activity distribution as for the ^18^F-PSMA, but corresponding to a whole-body activity of 6.5 MBq, equivalent to imaging 5 h post-administration of 10 MBq ^212^Pb-PSMA with an effective half-life of 8 h [5]. The activity concentrations with ^212^Pb are tabulated in Table 1.

**Table 1:** Accuracy of SPECT quantification for 30 × 2 reconstruction

	Volume [ml]	Image set	${a}_{gamma counter}$ [Bq/ml]	${\Delta }_{rel}$[%]							
				79 keV				239 keV			
				64 × 64		256 × 256		64 × 64		256 × 256	
Liver	2188	Set A	1155	− 27 ± 1		− 33 ± 1		− 37 ± 3		− 43 ± 1	
		Set B	324	− 18 ± 5		− 14 ± 2		4 ± 13		− 21 ± 1	
R. Kidney	170	Set A	1171	− 40 ± 5		− 40 ± 5		− 50 ± 8		− 46 ± 3	
		Set B	328	− 33 ± 14		− 25 ± 2		− 19 ± 16		− 28 ± 5	
L. Kidney	188	Set A	750	− 46 ± 2		− 45 ± 1		− 42 ± 2		− 49 ± 1	
		Set B	210	− 42 ± 4		− 25 ± 5		3 ± 9		− 22 ± 5	
Vertebral bodies	133	Set A	54	574 ± 75		741 ± 48		488 ± 88		743 ± 50	
Background	27,900	Set A	27	742 ± 20		968 ± 27		748 ± 23		1048 ± 29	

The mean ${\Delta }_{rel}$ and standard deviation of three SPECT images compared to ${a}_{gamma counter}$ for the two matrix sizes

### Imaging and reconstruction parameters

PET/CT imaging of the phantom was performed with a GE Discovery MI, using a 5-min acquisition over a single bed. Reconstruction was conducted with the system’s QCFX algorithm (otherwise known as “Q.Clear”), using attenuation correction by CT. The reconstructed volume consisted of 1.82 × 1.82 × 2.79 mm^3^ voxels.

PET imaging of the patient was performed with a GE Discovery 690, and 90 s per bed. Reconstruction employed the same QCFX (“Q.Clear”) algorithm with attenuation correction by CT. The reconstructed volume consisted of 3.27 × 2.73 × 2.73 mm^3^ voxels.

SPECT/CT imaging was performed on a Siemens Symbia Intevo Bold, with high-energy (HE) collimators. Regular QC and sensitivity tests have been performed. Two windows were captured; a 40% energy window centred at 79 keV with 20% adjacent dual scatter windows, and a 20% window centred at 239 keV with 5% adjacent dual scatter windows. The windows were reconstructed separately using a workflow with AutoRecon and Siemens’ Flash-3D, an OSEM-based vendor algorithm, with attenuation correction by CT and triple energy window scatter correction. Six combinations of iterations and subsets (*iterations* × *subsets*), 10 × 1, 15 × 1, 30 × 1, 30 × 2, 30 × 3, and 30 × 4, were used for each energy. An 8.4 mm Gaussian post-filter and a 12 mm Gaussian scatter-filter were applied. Matrix sizes of 256 × 256 and 64 × 64 were used.

Two sets of SPECT/CT images, set A and set B, were acquired at two different time points set approximately 19 h apart, with three 30-min acquisitions at each time point and an additional nine-hour acquisition in-between. For the first set of acquisitions, set A, the total activity in the phantom was 3.4–3.8 MBq, and for the later set B the total activity was 1.0–1.1 MBq. Additionally, seven SPECT/CT acquisitions of a homogeneous phantom with 1.2–7.9 MBq from a previous study [3] were reconstructed with the workflow with AutoRecon used for reconstructions of the anthropomorphic phantom to provide calibration factors (CFs).

### Quantitative analysis

Calibration factors were found from the same image acquisitions and methodology as in Kvassheim et al. [3], but reconstructed anew with the same reconstruction parameters and workflow as used with the anthropomorphic phantom. Activity measurements from the emission images were compared to 1 ml samples extracted from the phantom compartments and measured using an automatic gamma counter (Hidex AMG, Hidex Oy, Finland). Each compartment of the phantom was segmented in the CT-image and the SPECT mean voxel values within the segments were converted to activity concentrations, ${a}_{SPECT}$, by applying the CFs. Background was not subtracted. These were then compared to the activity concentrations measured on the gamma counter, ${a}_{gamma counter}$, according to

$${\Delta }_{rel}= \frac{{a}_{SPECT}-{a}_{gamma counter}}{{a}_{gamma counter}},$$

to provide a metric for the quantitative accuracy as the relative difference ${\Delta }_{rel}$. The background and vertebral bodies’ activity concentrations from the set B images were excluded, due to the low activity concentrations. All voxel values within the segmentations were extracted to study the distributions.

Recovery coefficients (RC) were also calculated for the kidney and liver volumes. These were taken as the ratio of the activity concentration in compartment volume in the SPECT images to the activity concentration as measured by gamma counter. The fractional uncertainty (FU) was propagated from the standard deviations of the mean counts in the compartment volumes in each image set and the standard deviations from the CFs.

## Results

### ***Verification of phantom setup and methodology with ***^***18***^***F***

**Fig. 1:**
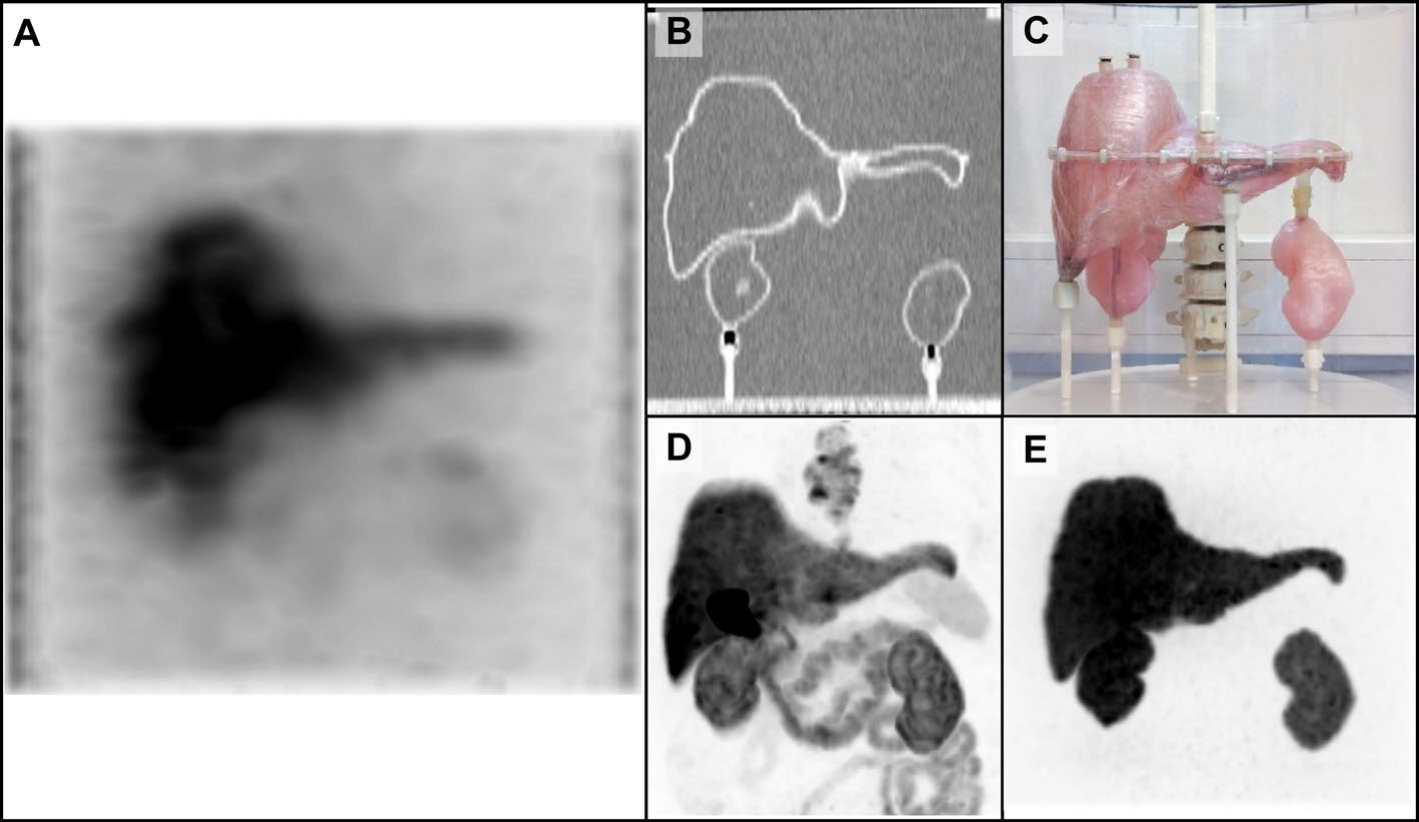
Images of the phantom and patient. (**A**) SPECT MIP of the phantom from a 9-h acquisition, with the total activity in the phantom starting at 3.0 MBq and decaying to 1.7 MBq, reconstructed as 30 × 1 with summed energy windows. (**B**) Coronal slice of a CT image of the phantom with 10 mm thick slab reconstruction. (**C**) Photo of the phantom. (**D**) Coronal MIP of the patient’s [^18^F]F-PSMA PET image. (**E**) Coronal MIP of the phantom ^18^F image

A maximum intensity projection (MIP) of the patient’s PET image can be seen in Fig. 1D. The PET image of the phantoms can be seen in Fig. 1E.

### Calibration factors

**Fig. 2:**
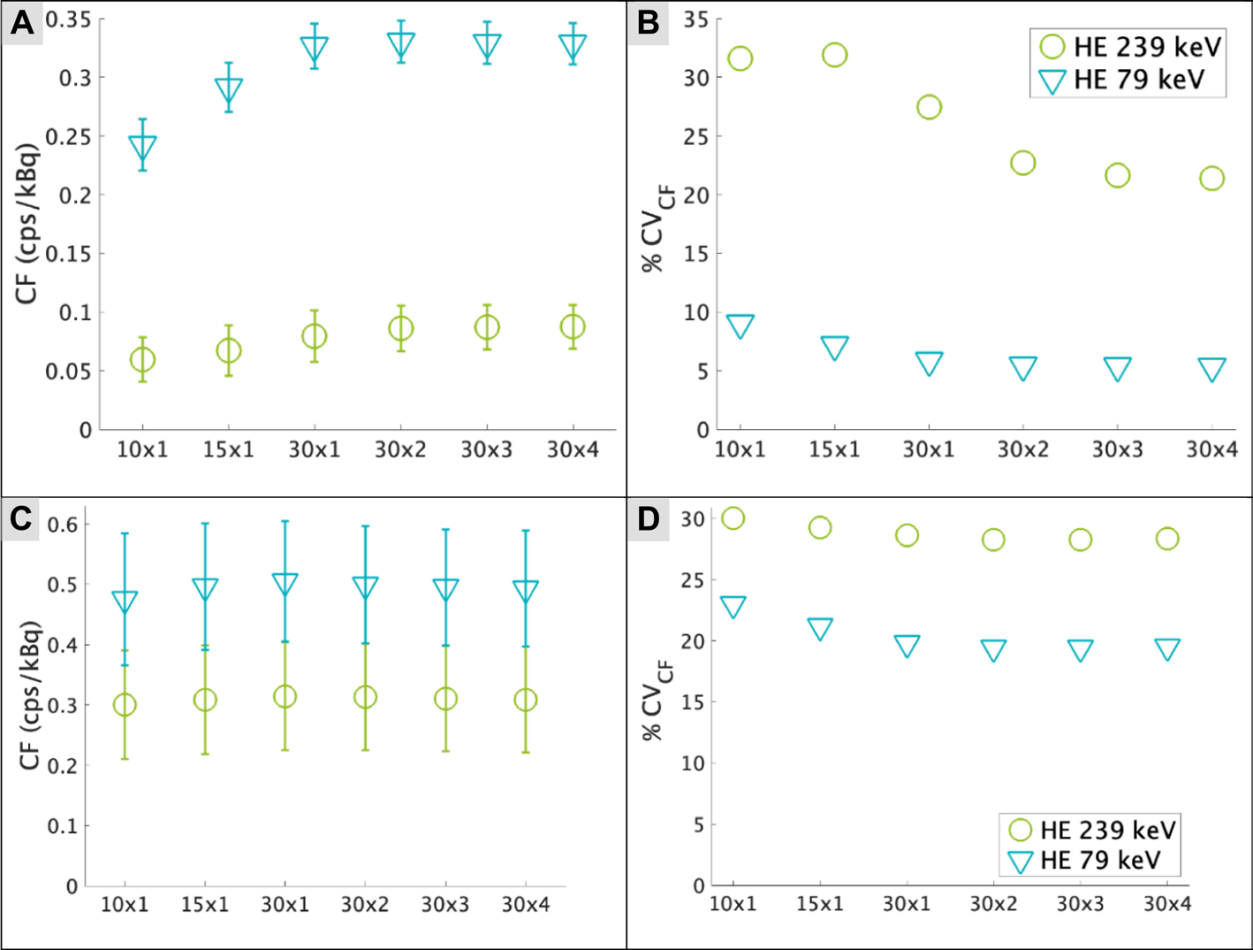
The calibration factors (CF) for the different reconstructions given with 64 × 64 (**A**) and 256 × 256 (**C**) matrices. Error bars represent the standard deviation between 7 acquisitions. The CV for the two energy windows and different reconstructions are shown for 64 × 64 (**B**) and 256 × 256 (**D**)

The means and standard deviations of the CFs for ^212^Pb for the 64 × 64 and 256 × 256 matrices are illustrated in Fig. 2, with its specific values tabulated in the supplementary material. The coefficients of variation (CV) are also given in Fig. 2.

### ***Quantitative accuracy of ***^***212***^***Pb SPECT imaging***

**Fig. 3:**
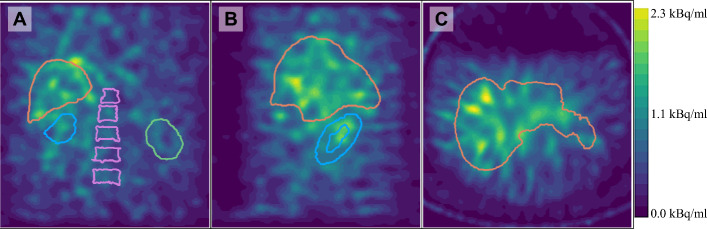
Example slices from a reconstruction used for quantitative analysis. (**A**) Coronal, (**B**) sagittal, and (**C**) axial slice of a SPECT image, with a total 3.8 MBq ^212^Pb in the phantom and 30 × 2 reconstructed with a 256 × 256 matrix and the 79 keV energy window. The liver, left kidney, right kidney, and vertebral bodies are outlined with orange, green, blue, and pink contours, respectively

**Fig. 4:**
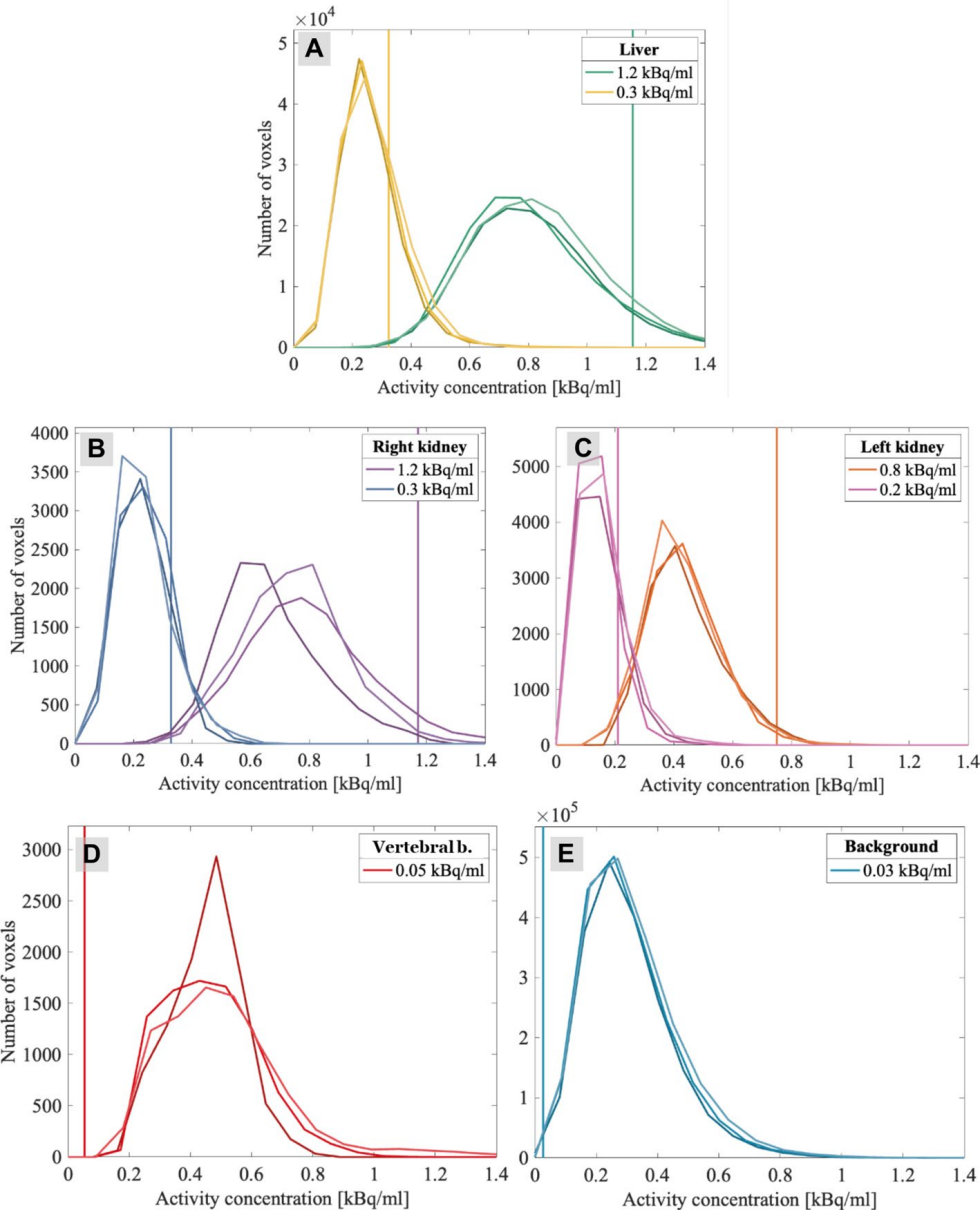
Histograms showing the number of voxels in each compartment corresponding to activity values. Phantom compartments of (**A**) liver, (**B**) right kidney, (**C**) left kidney, (**D**) vertebral bodies and (**E**) background are shown in separate panels. Each scan from the two image sets are plotted within each panel, with different colours for the two sets. The images were reconstructed with 30 × 2, using the 79 keV energy window, and the 256 × 256 matrix. The 239 keV energy window, and the 64 × 64 matrix results are given in the supplementary material. The vertical lines represent the activity concentrations as measured by gamma counter

The activity concentrations from SPECT were mostly smaller than gamma counter measurements for kidneys and liver, but larger for vertebral bodies and background (Table 1). Example slices of the first SPECT acquisition can be seen in Fig. 3. The liver compartment with an activity concentration of around 0.3 kBq/ml produced the best accuracy across the different parameters. The distribution of voxel values is shown in Fig. 4.

### Impact of reconstruction parameters

**Fig. 5:**
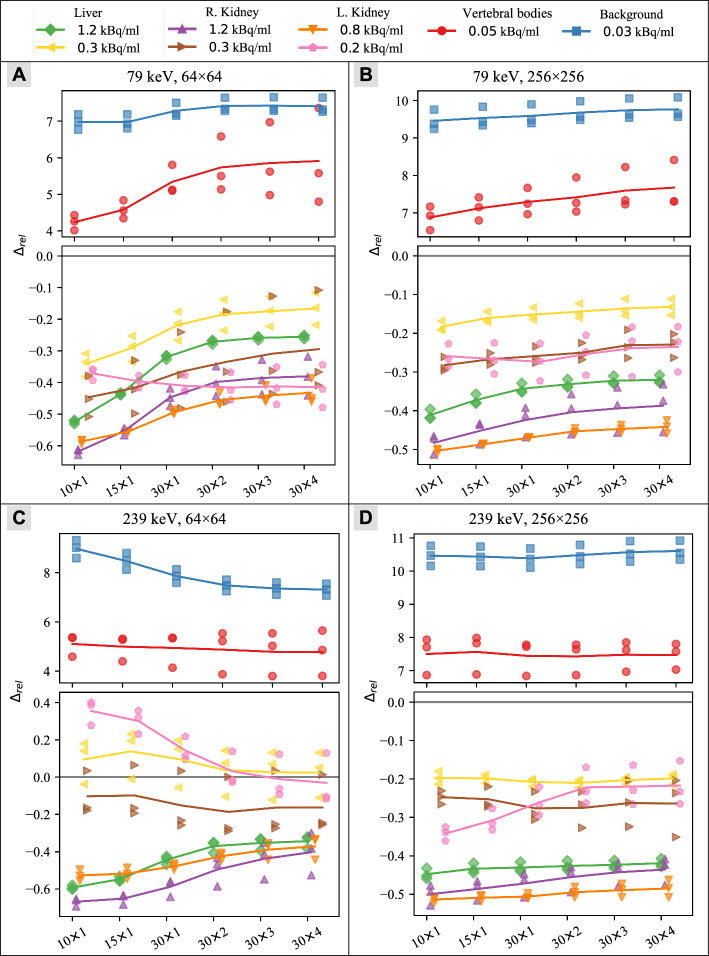
Quantitative accuracy by reconstruction parameters. The solid lines represents the mean of the three SPECT acquisitions with similar activity. The lines’ respective markers represents each SPECT acquisition. The 64 × 64 matrix results are shown in **A** for the 79 keV energy window and **C** for the 239 keV energy window. The 256 × 256 matrix results are shown in **B** for the 79 keV energy window and **D** for the 239 keV energy window. Every set of line and markers are slightly offset from other sets for better readability

Figure 5 shows the accuracy ${\Delta }_{rel}$ of each phantom compartment across different numbers of reconstruction updates, for both individual SPECT acquisitions and the sets’ mean values. Disregarding the very low activity concentrations of the background and vertebral bodies, the tendency is for the activity concentration to be underestimated.

### Recovery coefficients (RCs)

Table 2 presents RCs and fractional uncertainties, for the liver and kidneys for the two activity concentration levels. The RCs varied the least between activity levels for the 79 keV peak with the 64 × 64 matrix. The fractional uncertainties were also lowest with these settings.

**Table 2:** RCs and fractional uncertanties (FU)

30 × 2		64 × 64						256 × 256					
		79 keV			239 keV			79 keV			239 keV		
		RC	FU		RC	FU		RC	FU		RC	FU	
Liver	Set A	0.69	8 %		0.60	24 %		0.63	20 %		0.54	28 %	
	Set B	0.77	5 %		0.98	24 %		0.89	20 %		0.82	28 %	
R. kidney	Set A	0.57	9 %		0.48	26 %		0.56	20 %		0.51	28 %	
	Set B	0.62	17 %		0.77	31 %		0.78	20 %		0.76	30 %	
L. kidney	Set A	0.52	11 %		0.54	24 %		0.52	21 %		0.48	29 %	
	Set B	0.55	12 %		0.97	24 %		0.78	20 %		0.81	29 %	

All values are based on measurements from 30 × 2 reconstructions

## Discussion

To explore ^212^Pb quantitative imaging at low activity levels, we conducted a phantom study using patient-derived geometries. Quantification from SPECT images deviated from gamma counter measurements, but with activity concentrations ≥ 210 Bq/ml accuracies of less than 50% were achievable for most acquisition and reconstruction settings. In general, the RCs depended somewhat on activity level for most of the settings, and implementation of these to increase the accuracy should be performed with caution. However, the un-corrected results still indicate the potential for clinical quantification of ^212^Pb in patients.

The SPECT images of the phantom with ^212^Pb (Fig. 3) depict the liver clearly. Both kidneys can be vaguely identified but were more easily distinguished through measurements from the images. The vertebral bodies could not be visually distinguished from the background, but SPECT measurements on average indicated a contrast to background being positive and about half as large as the contrast indicated by gamma counter measurements. When considering only the background water in the immediate vicinity, a positive contrast was still consistently maintained.

SPECT quantifications mostly underestimated activity concentrations ≥ 210 Bq/ml (Figs. 4, 5), as could be expected due to intensity blurring. With ^177^Lu in an anthropomorphic kidney phantom, Salvadori et al. found 66–80% of the known activity with different target to background ratios [8] in their kidney segmentations without correcting for intensity blurring. With the inherently more challenging imaging properties of ^212^Pb, we expect intensity blurring is the main contributor to the systematic underestimation of activity. This is reflected in Table 2, showing that we find 52–97% of the known activities, but the fractional uncertainties stay around 20%.

However, the underestimation was slightly smaller for the set B images and seemed to change more with activity concentration than geometry, as ${\Delta }_{rel}$ was more consistent between compartments in the same set (Fig. 5). We think this may be explained by the skewed voxel intensity distributions seen in Fig. 4. For higher activity concentrations, the histograms are more symmetric, as they are not capped at zero. This might have been different if the reconstruction included negative values. The larger voxels of the 64 × 64 matrix will accumulate more counts, and hence the cap at zero is not as significant. This may explain why the RCs for the 64 × 64 matrix are more consistent between activity levels.

The deviations in quantification due to intensity blurring, could potentially be accounted for with RCs for kidneys and livers (Table 2). Hence, the variability of the three repeated acquisitions at the same activity levels may be better indicators of quantitative accuracy. The uncertainty on the RCs shown in Table 2 are a combination of the uncertainties of the CFs and the variation between images within a set, which can be studied in Fig. 5. For most of these fractional uncertainties (FU), the uncertainty of the CF is the major contributor. Generally the FUs are around 20%, which may be usable for quantification, however, the variation in RC with activity level emphasises the need for caution. As activity concentrations reach very low levels, the matrix considered for quantification of images should be based on the energy window; specifically, a 64 × 64 matrix with a 79 keV window yields the most consistent RC across activity levels.

Kvassheim et al. suggested a minimum of 160 Bq/ml for reliable CFs [3], which our results align with. The quantitative deviations in activity concentrations in the vertebral bodies (52–58 Bq/ml) and the background (26–29 Bq/ml) were too large to allow reasonable quantification, while the low-activity measurement of the left kidney (210 Bq/ml) and all measurements with higher activity concentrations had less than 50% deviation.

The total activity imaged in the phantom was 1.0–3.8 MBq ^212^Pb, which corresponded to an estimated whole-body activity of 1.7–6.5 MBq in the patient. The distribution and activity level of ^212^Pb in the phantom was simulated to correspond to 5.0 h post-injection of 10 MBq ^212^Pb-PSMA, assuming the distribution would be the same as in ^18^F-PSMA PET image of the patient and an 8 h effective whole-body half-life as found in a phase 0 trial of [^212^Pb]Pb-PSMA (AB001) therapy [5]. Without tissue-specific information, the final measurement time point for dosimetry could be around 5 times the effective half-life [9], corresponding to 40 h post-injection for ^212^Pb-PSMA [5]. If 60 MBq ^212^Pb is administered to a patient, as in a recently published case study [10], an estimated 2 MBq would remain in the patient after 40 h [5]. Our results show then, that quantitative imaging 40 h post administration for dosimetry calculations may be feasible for larger organs such as kidneys and livers with  ≥ 210 Bq/ml ^212^Pb, though with some considerable uncertainties. Earlier post-therapy imaging comes with other challenges, as Kästner et al. [11] reported that deadtime effects with an estimated 20% count loss from approximately 40 MBq ^212^Pb when using HE collimator.

## Conclusion

This study demonstrates promising trends across multiple imaging parameters for quantification of low activity concentrations ( ≥ 210 Bq/ml) by SPECT imaging of ^212^Pb using an anthropomorphic phantom. Intensity blurring contributes to underestimations, and to further improve the quantification potential in a clinical setting, development to characterise how RCs change with activity concentration, or to apply different correction methods, are necessary. Still, the consistency of measurement suggest potential for patient quantification. Depending on the application, the observed uncertainties of low ^212^Pb activity imaging may even be acceptable, for instance for late imaging time point quantification for dosimetry.

## Supplementary Information

Supplementary file 1.

## Data Availability

The data used and/or analysed are available on reasonable request.

## Abbreviations

CF: Calibration factor
CT: Computed tomography
CV: Coefficient of variation
MIP: Maximum intensity projection
OSEM: Ordered subset expectation maximisation
PET: Positron emission tomography
PLA: Polylactic acid
PSMA: Prostate specific membrane antigen
SPECT: Single photon emission computed tomography
TAT: Targeted alpha therapy
TEW: Triple energy window scatter correction
TRT: Targeted radionuclide therapy
^212^Bi: Bismuth-212
^18^F: Flourine-18
^177^Lu: Lutetium-177
^212^Pb: Lead-212
