# Supplementary material for: Assessing the feasibility of quantitative SPECT imaging for low 212Pb activity concentrations using anthropomorphic phantoms
Source: EJNMMI Phys. 2026 Jan 5;13:42. doi: 10.1186/s40658-025-00829-1 (PMC13157375; doi:10.1186/s40658-025-00829-1)
Supplement: Supplementary file 1 [file 40658_2025_829_MOESM1_ESM.pdf]

Assessing the feasibility of quantitative SPECT imaging for low ^{212}Pb activity concentrations using anthropomorphic phantoms

Johan Høiness[1,2]*, Caroline Stokke[1,2], Eivor Hernes[3], Lars Tore Gyland Mikalsen[2], Monika Kvassheim[2,4]

[1]Department of Physics, University of Oslo, Norway
[2]Department of Physics and Computational Radiology, Division of Radiology and Nuclear Medicine, Oslo University Hospital, Oslo, Norway
[3]Department of Nuclear Medicine, Division of Radiology and Nuclear Medicine, Oslo University Hospital, Oslo, Norway
[4]Faculty of Medicine, University of Oslo, Oslo, Norway
(*) Corresponding author: j.s.hoiness@gmail.com

Supplementary material

Table 1: CFs with a 64×64 and 256×256 matrices.

	CF (cps/kBq) with 79 keV window		CF (cps/kBq) with 239 keV window	
	64×64 matrix	256×256 matrix	64×64 matrix	256×256 matrix
10×1	0.242 ± 0.022	0.475 ± 0.109	0.059 ± 0.019	0.300 ± 0.090
15×1	0.292 ± 0.021	0.496 ± 0.105	0.067 ± 0.021	0.308 ± 0.090
30×1	0.327 ± 0.019	0.505 ± 0.100	0.080 ± 0.022	0.314 ± 0.090
30×2	0.330 ± 0.018	0.499 ± 0.097	0.086 ± 0.020	0.313 ± 0.088
30×3	0.329 ± 0.018	0.495 ± 0.096	0.087 ± 0.019	0.310 ± 0.088
30×4	0.329 ± 0.018	0.493 ± 0.096	0.087 ± 0.019	0.309 ± 0.088

Calibration factors (CF) are given with the mean and standard deviations of 7 acquisitions with 1.2-7.9 MBq ^{212}Pb in the phantom.

Table 2: Accuracy of SPECT quantification for 10×1 reconstruction.

	Volume [ml]	Image set	$a_{gamma\ counter}$ [Bq/ml]	$\%\Delta_{rel}$ [%] 79 keV		239 keV	
				64×64	256×256	64×64	256×256
Liver	2188	Set A	1155	− 52 ± 1	− 41 ± 1	− 59 ± 1	− 45 ± 1
		Set B	324	− 34 ± 4	− 18 ± 1	9 ± 12	− 20 ± 2
R. Kidney	170	Set A	1171	− 62 ± 1	− 48 ± 3	− 67 ± 2	− 50 ± 3
		Set B	328	− 45 ± 6	− 28 ± 2	− 10 ± 12	− 25 ± 2
L. Kidney	188	Set A	750	− 59 ± 1	− 50 ± 1	− 53 ± 3	− 51 ± 1
		Set B	210	− 37 ± 2	− 26 ± 3	36 ± 7	− 34 ± 2
Vertebral bodies	133	Set A	54	423 ± 21	688 ± 32	511 ± 45	750 ± 56
Background	27900	Set A	27	698 ± 21	946 ± 27	897 ± 36	1046 ± 30

The mean $\%\Delta_{rel}$ and standard deviation of three SPECT images compared to $a_{gamma\ counter}$ for the two matrix sizes.

Table 3: Accuracy of SPECT quantification for 15×1 reconstruction.

	Volume [ml]	Image set	$a_{gamma\ counter}$ [Bq/ml]	$\%\Delta_{rel}$ [%] 79 keV		239 keV	
				64×64	256×256	64×64	256×256
Liver	2188	Set A	1155	− 44 ± 0	− 37 ± 1	− 55 ± 1	− 43 ± 1
		Set B	324	− 29 ± 4	− 16 ± 1	14 ± 13	− 20 ± 1
R. Kidney	170	Set A	1171	− 55 ± 1	− 45 ± 3	− 65 ± 3	− 49 ± 3
		Set B	328	− 42 ± 8	− 27 ± 2	− 10 ± 14	− 25 ± 3
L. Kidney	188	Set A	750	− 56 ± 0	− 49 ± 0	− 52 ± 3	− 51 ± 1
		Set B	210	− 40 ± 2	− 27 ± 4	30 ± 6	− 31 ± 3
Vertebral bodies	133	Set A	54	458 ± 25	712 ± 31	500 ± 52	756 ± 59
Background	27900	Set A	27	697 ± 19	953 ± 26	847 ± 33	1044 ± 29

The mean $\%\Delta_{rel}$ and standard deviation of three SPECT images compared to $a_{gamma\ counter}$ for the two matrix sizes.

Table 4: Accuracy of SPECT quantification for 30×1 reconstruction.

	Volume [ml]	Image set	$a_{gamma\ counter}$ [Bq/ml]	$\%\Delta_{rel}$ [%] 79 keV		239 keV	
				64×64	256×256	64×64	256×256
Liver	2188	Set A	1155	– 32 ± 1	– 34 ± 1	– 44 ± 2	– 43 ± 1
		Set B	324	– 22 ± 5	– 15 ± 2	10 ± 14	– 21 ± 1
R. Kidney	170	Set A	1171	– 45 ± 3	– 42 ± 4	– 59 ± 5	– 47 ± 3
		Set B	328	– 37 ± 12	– 26 ± 2	– 15 ± 16	– 28 ± 4
L. Kidney	188	Set A	750	– 49 ± 1	– 47 ± 0	– 48 ± 2	– 51 ± 1
		Set B	210	– 41 ± 3	– 27 ± 5	14 ± 7	– 26 ± 4
Vertebral bodies	133	Set A	54	534 ± 40	729 ± 35	494 ± 70	745 ± 53
Background	27900	Set A	27	728 ± 19	959 ± 27	786 ± 27	1038 ± 29

The mean $\%\Delta_{rel}$ and standard deviation of three SPECT images compared to $a_{gamma\ counter}$ for the two matrix sizes.

Table 5: Accuracy of SPECT quantification for 30×2 reconstruction.

	Volume [ml]	Image set	$a_{gamma\ counter}$ [Bq/ml]	$\%\Delta_{rel}$ [%] 79 keV		239 keV	
				64×64	256×256	64×64	256×256
Liver	2188	Set A	1155	– 27 ± 1	– 33 ± 1	– 37 ± 3	– 43 ± 1
		Set B	324	– 18 ± 5	– 14 ± 2	4 ± 13	– 21 ± 1
R. Kidney	170	Set A	1171	– 40 ± 5	– 40 ± 5	– 50 ± 8	– 46 ± 3
		Set B	328	– 33 ± 14	– 25 ± 2	– 19 ± 16	– 28 ± 5
L. Kidney	188	Set A	750	– 46 ± 2	– 45 ± 1	– 42 ± 2	– 49 ± 1
		Set B	210	– 42 ± 4	– 25 ± 5	3 ± 9	– 22 ± 5
Vertebral bodies	133	Set A	54	574 ± 75	741 ± 48	488 ± 88	743 ± 50
Background	27900	Set A	27	742 ± 20	968 ± 27	748 ± 23	1048 ± 29

The mean $\%\Delta_{rel}$ and standard deviation of three SPECT images compared to $a_{gamma\ counter}$ for the two matrix sizes.

Table 6: Accuracy of SPECT quantification for 30×3 reconstruction.

	Volume [ml]	Image set	$a_{gamma\ counter}$ [Bq/ml]	$\%\Delta_{rel}$ [%] 79 keV 64×64	$\%\Delta_{rel}$ [%] 79 keV 256×256	$\%\Delta_{rel}$ [%] 239 keV 64×64	$\%\Delta_{rel}$ [%] 239 keV 256×256
Liver	2188	Set A	1155	– 26 ± 1	– 32 ± 1	– 35 ± 3	– 42 ± 1
		Set B	324	– 17 ± 5	– 14 ± 2	3 ± 13	– 20 ± 1
R. Kidney	170	Set A	1171	– 39 ± 6	– 39 ± 6	– 44 ± 10	– 44 ± 3
		Set B	328	– 31 ± 16	– 23 ± 4	– 16 ± 17	– 26 ± 6
L. Kidney	188	Set A	750	– 44 ± 3	– 45 ± 1	– 39 ± 5	– 49 ± 2
		Set B	210	– 41 ± 6	– 24 ± 7	– 1 ± 12	– 22 ± 5
Vertebral bodies	133	Set A	54	586 ± 101	759 ± 55	479 ± 89	748 ± 46
Background	27900	Set A	27	742 ± 20	974 ± 28	735 ± 25	1057 ± 31

The mean $\%\Delta_{rel}$ and standard deviation of three SPECT images compared to $a_{gamma\ counter}$ for the two matrix sizes.

Table 7: Accuracy of SPECT quantification for 30×4 reconstruction.

	Volume [ml]	Image set	$a_{gamma\ counter}$ [Bq/ml]	$\%\Delta_{rel}$ [%] 79 keV 64×64	$\%\Delta_{rel}$ [%] 79 keV 256×256	$\%\Delta_{rel}$ [%] 239 keV 64×64	$\%\Delta_{rel}$ [%] 239 keV 256×256
Liver	2188	Set A	1155	– 26 ± 1	– 32 ± 1	– 34 ± 3	– 42 ± 1
		Set B	324	– 17 ± 5	– 13 ± 2	2 ± 12	– 20 ± 2
R. Kidney	170	Set A	1171	– 38 ± 6	– 39 ± 6	– 40 ± 11	– 44 ± 4
		Set B	328	– 29 ± 16	– 23 ± 3	– 16 ± 15	– 26 ± 8
L. Kidney	188	Set A	750	– 43 ± 4	– 44 ± 2	– 37 ± 6	– 48 ± 2
		Set B	210	– 41 ± 7	– 24 ± 6	– 3 ± 14	– 22 ± 6
Vertebral bodies	133	Set A	54	591 ± 131	767 ± 64	477 ± 92	747 ± 39
Background	27900	Set A	27	741 ± 21	976 ± 28	731 ± 24	1061 ± 29

The mean $\%\Delta_{rel}$ and standard deviation of three SPECT images compared to $a_{gamma\ counter}$ for the two matrix sizes.

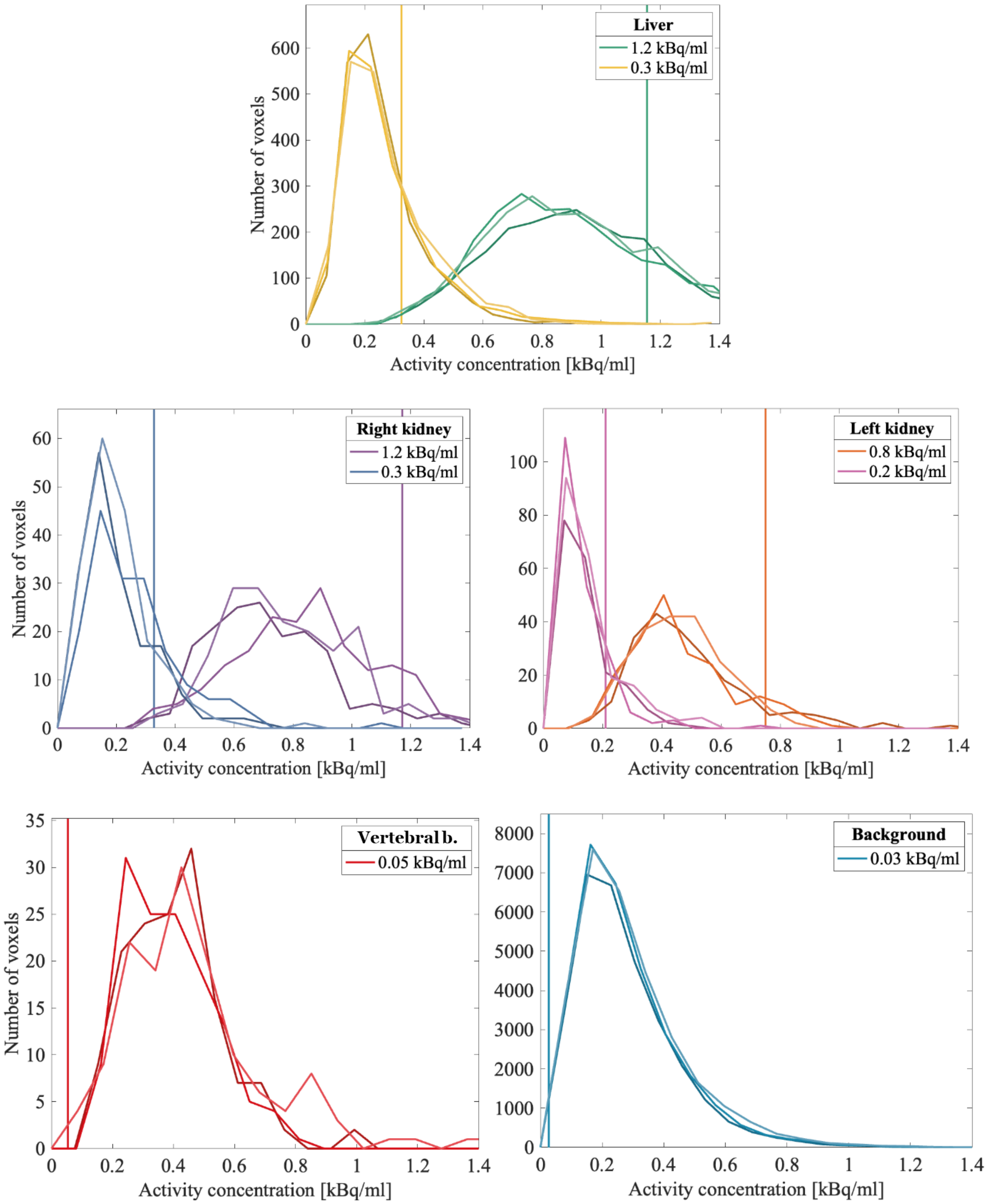

Figure 1: Histograms showing the number of voxels in each compartment corresponding to activity values. Each phantom compartment is shown in a panel, and the two image sets are plotted in different colours. The vertical lines represent the expected activity concentrations as measured by gamma counter. The images were reconstructed with 30×2, using the 79 keV energy window, and the 64×64 matrix.

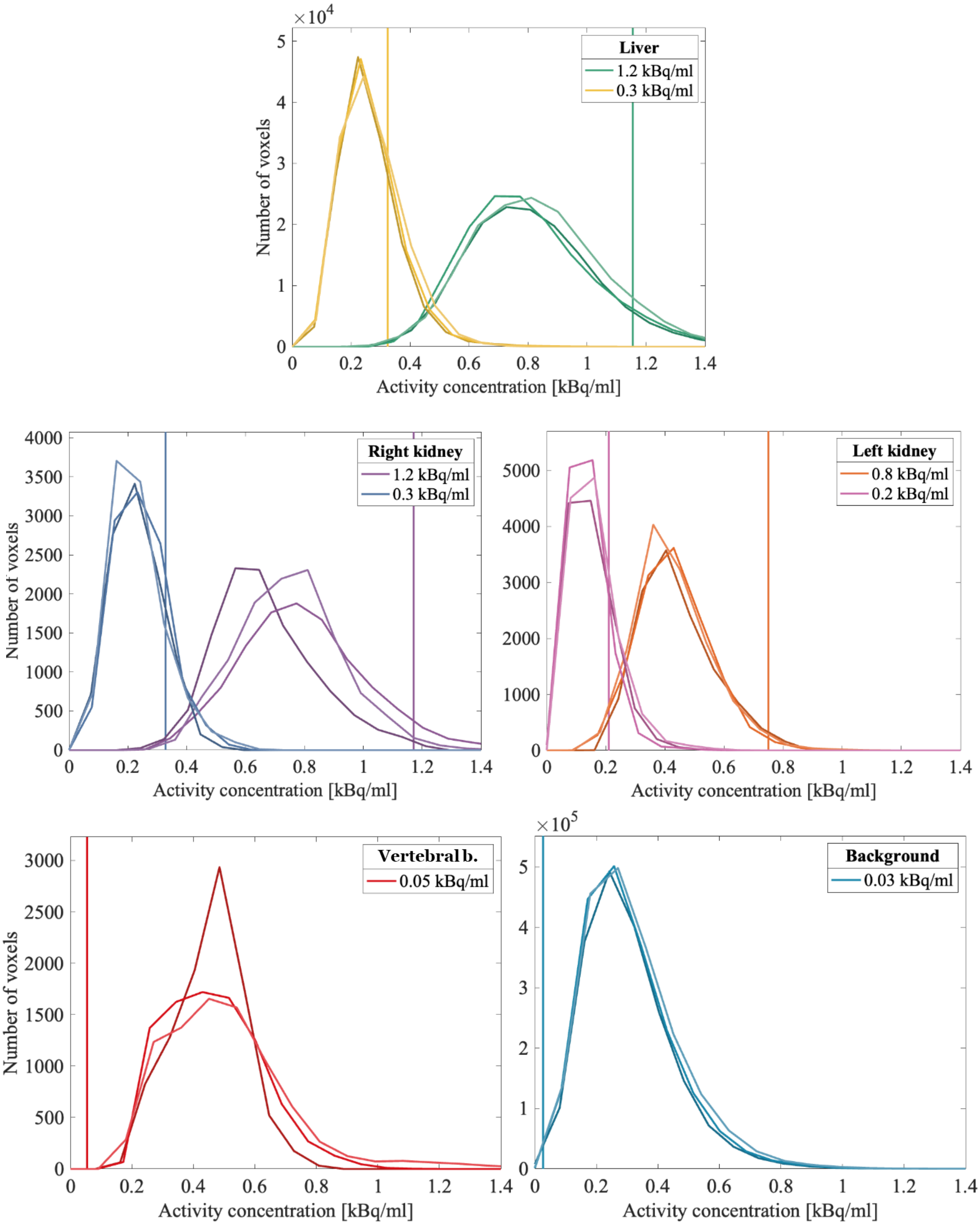

Figure 2: Histograms showing the number of voxels in each compartment corresponding to activity values. Each phantom compartment is shown in a panel, and the two image sets are plotted in different colours. The vertical lines represent the expected activity concentrations as measured by gamma counter. The images were reconstructed with 30×2, using the 79 keV energy window, and the 256×256 matrix.

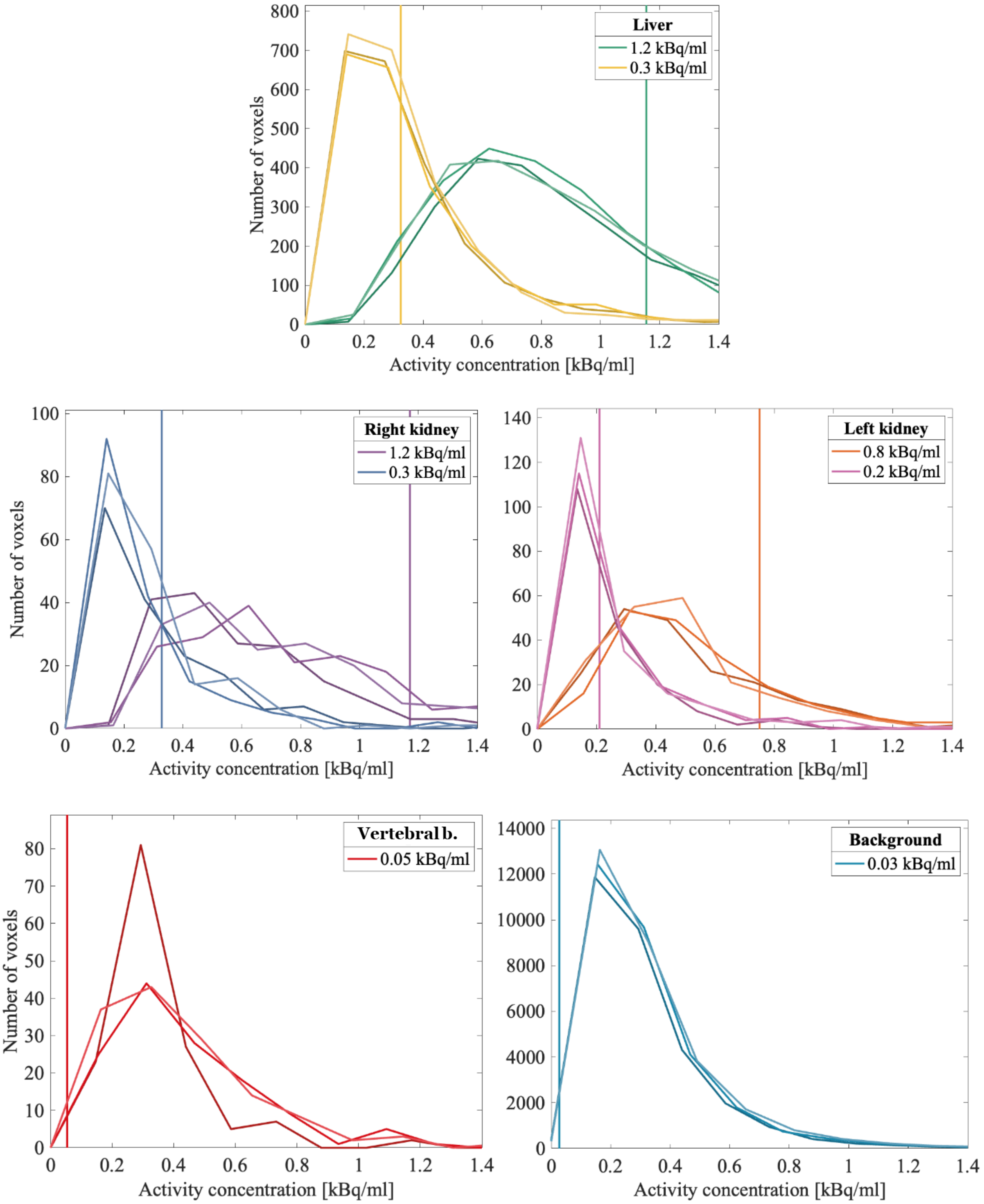

Figure 3: Histograms showing the number of voxels in each compartment corresponding to activity values. Each phantom compartment is shown in a panel, and the two image sets are plotted in different colours. The vertical lines represent the expected activity concentrations as measured by gamma counter. The images were reconstructed with 30×2, using the 239 keV energy window, and the 64×64 matrix.

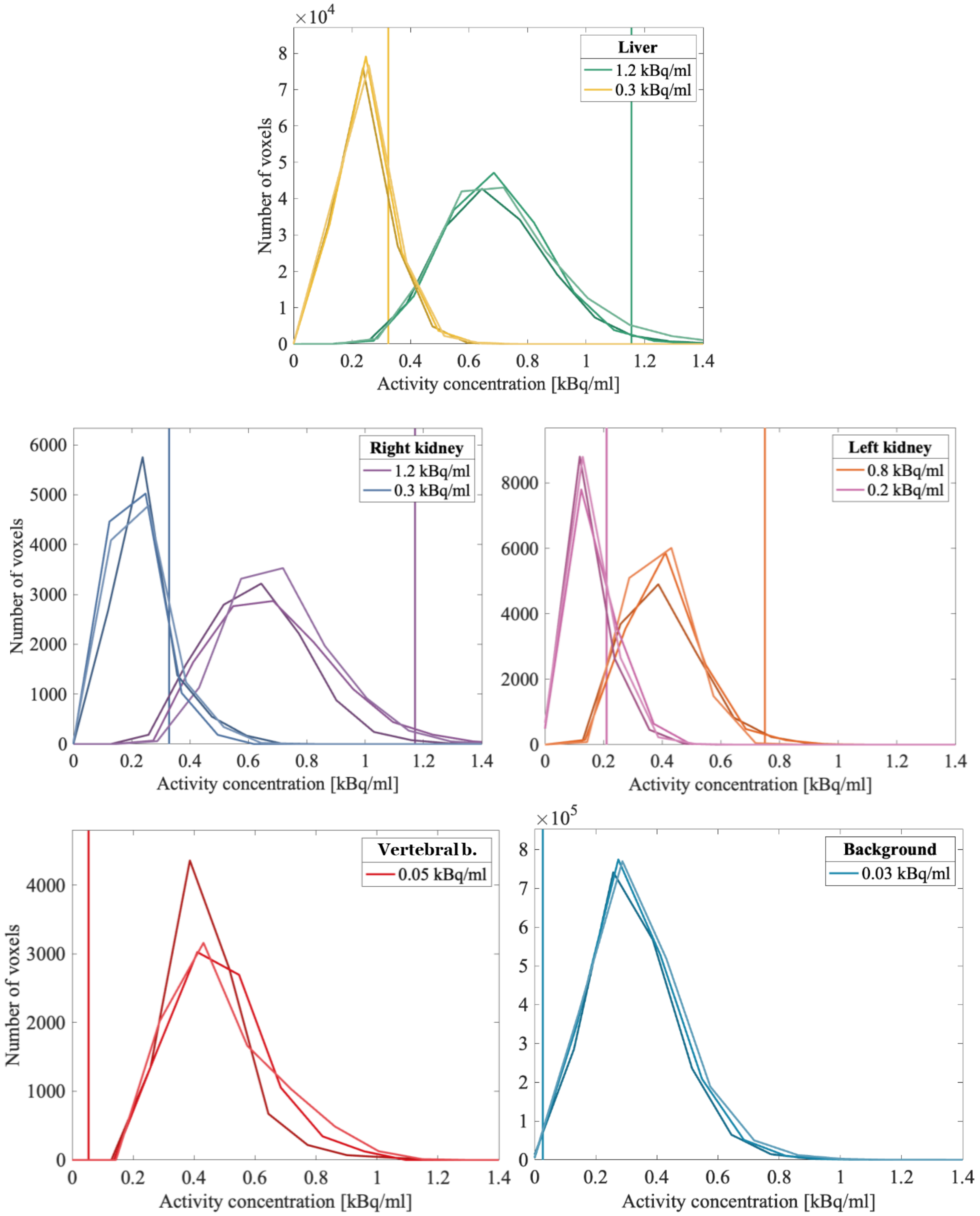

Figure 4: Histograms showing the number of voxels in each compartment corresponding to activity values. Each phantom compartment is shown in a panel, and the two image sets are plotted in different colours. The vertical lines represent the expected activity concentrations as measured by gamma counter. The images were reconstructed with 30×2, using the 239 keV energy window, and the 256×256 matrix.
